# Inflammatory Load Across Diabetes Duration: CRP and ESR Patterns and Their Metabolic Correlates

**DOI:** 10.3390/metabo16030202

**Published:** 2026-03-19

**Authors:** Roxana Daniela Brata, Cosmin Mihai Vesa, Madalina Ioana Moisi, Timea Claudia Ghitea, Nicolae Ovidiu Pop, Carmen Pantis

**Affiliations:** 1 Department of Clinical Discipline, Faculty of Medicine and Pharmacy, University of Oradea, 410068 Oradea, Romania; 2 Clinical County Emergency Hospital Bihor, 410169 Oradea, Romania; 3 Doctoral School of Biomedical Sciences, Faculty of Medicine and Pharmacy, University of Oradea, 410087 Oradea, Romania; 4 Department of Preclinical Discipline, Faculty of Medicine and Pharmacy, University of Oradea, 410068 Oradea, Romaniapop.nicolaeovidiu@didactic.uoradea.ro (N.O.P.); carmen.pantis@didactic.uoradea.ro (C.P.); 5 Pharmacy Department, Faculty of Medicine and Pharmacy, University of Oradea, 410068 Oradea, Romania

**Keywords:** type 2 diabetes, systemic inflammation, C-reactive protein, erythrocyte sedimentation rate, diabetes duration, chronic kidney disease, cardio–renal axis

## Abstract

**Highlights:**

**What are the main findings?**

- Inflammatory burden in type 2 diabetes follows a nonlinear cross-sectional pattern across disease duration, with elevated CRP in early metabolic stages and in long-standing disease.
- CRP correlates significantly with adiposity (BMI) and atherogenic dyslipidemia (triglyceride-to-HDL ratio), but not with HbA1c, suggesting that inflammation reflects metabolic phenotype rather than short-term glycemic control.

**What are the implications of the main findings?**

- Systemic inflammation in T2DM appears to be driven by adipose–lipid metabolic dysfunction in early disease and by organ damage (particularly renal impairment) in advanced stages.
- Targeting adiposity and dyslipidemia may represent a key strategy for modulating inflammatory load beyond glucose-centered management.

**Abstract:**

Background: Type 2 diabetes mellitus (T2DM) is characterized by chronic low-grade inflammation that contributes to cardiometabolic complications. While diabetes duration reflects cumulative metabolic exposure, its relationship with systemic inflammatory burden remains insufficiently defined. We aimed to investigate inflammatory patterns across diabetes duration and to explore their metabolic and cardio–renal correlates. Methods: This real-world cross-sectional study included 250 adults with T2DM. Diabetes duration was analyzed both continuously and across four predefined strata (0–4, 5–9, 10–14, and ≥15 years). Inflammatory burden was assessed using C-reactive protein (CRP) and erythrocyte sedimentation rate (ESR). Given the skewed distribution of CRP, log-transformed CRP was used in regression analyses. Nonlinear associations were evaluated using quadratic regression models. This approach was selected because preliminary descriptive analyses suggested a non-monotonic relationship between diabetes duration and CRP levels. Inclusion of a quadratic term allowed formal testing of a potential curvilinear association between diabetes duration and inflammatory burden. Spearman correlations were performed to assess associations with metabolic, renal, and cardiovascular variables. Results: CRP showed a nonlinear cross-sectional association across diabetes duration strata. Median CRP values were higher in early (0–4 years: 0.62 mg/L) and long-standing diabetes (≥15 years: 0.77 mg/L) compared with intermediate-duration groups (*p* = 0.063). Quadratic regression confirmed a U-shaped relationship (adjusted β_duration = −0.079, *p* < 0.001; β_duration^2^ = 0.0027, *p* < 0.001; R^2^ = 0.326). ESR differed significantly across duration strata (*p* = 0.002), with the highest levels observed in long-standing diabetes. CRP correlated positively with BMI (ρ = 0.151; *p* = 0.017) and triglyceride-to-HDL ratio (ρ = 0.215; *p* < 0.001), but not with HbA1c. Both CRP and ESR were more strongly associated with functional CKD (ρ = 0.350 and 0.429, respectively; *p* < 0.001) than with ASCVD. Conclusions: Inflammatory burden in T2DM shows a nonlinear cross-sectional pattern across diabetes duration, characterized by elevated levels in early and long-standing disease. Systemic inflammation appears more closely linked to renal dysfunction than to established cardiovascular disease. These findings support a cardio–renal–inflammatory axis in which prolonged diabetes exposure contributes to renal decline, which in turn amplifies systemic inflammatory activation.

## 1. Introduction

Type 2 diabetes mellitus (T2DM) is increasingly recognized not only as a metabolic disorder characterized by chronic hyperglycemia but also as a state of persistent low-grade systemic inflammation. Inflammatory activation plays a central role in the pathogenesis of insulin resistance, endothelial dysfunction, atherosclerosis, and diabetic microvascular complications. Elevated levels of inflammatory markers such as CRP and ESR have been associated with adverse cardiovascular and renal outcomes. These markers were selected because they are widely available and routinely measured in clinical practice, allowing assessment of systemic inflammatory burden in real-world cohorts. However, both CRP and ESR are nonspecific indicators of inflammation and do not capture the full complexity of inflammatory pathways involved in diabetes [1,2,3].

The inflammatory profile of T2DM is complex and dynamic. Early in the disease course, inflammation is largely driven by adipose tissue dysfunction, macrophage infiltration, and cytokine production linked to insulin resistance. As the disease progresses, cumulative metabolic stress and organ damage may further amplify systemic inflammation. However, the cross-sectional variation in inflammatory burden across different diabetes-duration strata remains insufficiently characterized in real-world clinical cohorts. While inflammatory markers have been widely studied in type 2 diabetes, relatively few studies have explored how systemic inflammation differs across stages of disease duration while simultaneously considering metabolic, renal, and cardiovascular correlates [4,5,6,7].

Diabetes duration is commonly used as a surrogate marker of cumulative metabolic exposure and is strongly associated with microvascular and macrovascular complications. Yet it remains unclear whether inflammatory markers increase progressively with longer disease duration or whether more complex, nonlinear patterns emerge over time. Identifying such patterns may improve understanding of disease staging and multisystem involvement in T2DM [8,9,10,11].

Moreover, inflammation may be differentially linked to cardiovascular and renal complications. While chronic inflammation is a recognized contributor to atherosclerosis, renal dysfunction itself can act as a potent amplifier of systemic inflammation through impaired clearance of pro-inflammatory mediators and accumulation of uremic toxins. Disentangling the relative contributions of cardiovascular disease and chronic kidney disease (CKD) to inflammatory burden in diabetes may provide insight into underlying pathophysiological pathways [12,13,14].

In addition, metabolic parameters such as obesity, dyslipidemia, and glycemic control may differentially influence inflammatory activation. It remains uncertain whether inflammatory burden in T2DM is more closely associated with current glycemic status or with broader cardiometabolic phenotype and organ damage.

Therefore, the aim of the present study was to investigate the relationship between diabetes duration and systemic inflammatory burden in a real-world cohort of adults with T2DM. Specifically, we sought to (1) compare CRP and ESR levels across predefined duration strata; (2) evaluate potential nonlinear associations between diabetes duration and CRP using quadratic regression modeling; (3) explore metabolic and renal correlates of inflammatory markers; and (4) examine cardio–renal–inflammatory interactions within this population.

## 2. Materials and Methods

### 2.1. Study Design and Participants

This study represents a real-world, cross-sectional analysis conducted in adult patients with type 2 diabetes mellitus (T2DM) followed at Bihor County Emergency Clinical Hospital, Oradea, Romania. The objective was to evaluate the inflammatory burden across diabetes duration and to explore its metabolic and cardio-renal correlates.

Eligible participants were adults aged 18–80 years with a confirmed diagnosis of T2DM according to American Diabetes Association (ADA) criteria. All patients underwent standardized clinical and laboratory evaluation between March 2024 and September 2025. Patients with incomplete inflammatory or renal data were excluded. Individuals with acute infectious or inflammatory conditions at the time of evaluation were not included. Exclusion was based on clinical records, including evidence of acute infection, recent antibiotic use, leukocytosis, or markedly elevated CRP levels suggestive of acute inflammatory processes.

A total of 250 patients with complete data on diabetes duration and inflammatory parameters were included in the final analysis. All participants provided informed consent for the use of anonymized clinical data for research purposes.

The study was conducted in accordance with the Declaration of Helsinki and approved by the Institutional Review Board of the Faculty of Medicine and Pharmacy, University of Oradea (approval no. 5/30 October 2023).

### 2.2. Assessment of Inflammatory Markers

Systemic inflammatory burden was assessed using:

- C-reactive protein (CRP, mg/L).
- Erythrocyte sedimentation rate (ESR; mm/h).

CRP and ESR values were obtained from venous blood samples collected under standardized conditions. Given the skewed distribution of CRP values, logarithmic transformation [log(CRP + 1)] was applied for regression modeling. CRP measurements were obtained using a high-sensitivity immunoturbidimetric assay (hs-CRP) performed in the hospital’s central laboratory on an automated analyzer, Cobas 8000 (Roche Diagnostics, Mannheim, Germany). The assay had a lower limit of detection of approximately 0.1 mg/L and analytical coefficients of variation below 5% within the clinically relevant range.

Diabetes duration was recorded in years based on the documented date of diagnosis and analyzed both as a continuous variable and across four predefined strata:

- 0–4 years.
- 5–9 years.
- 10–14 years.
- ≥15 years.

To minimize confounding by acute inflammatory conditions, patients with clinical signs of infection, recent antibiotic treatment, leukocytosis, or CRP values suggestive of acute inflammation (>10 mg/L) were excluded whenever such data were available in the medical record.

### 2.3. Clinical and Cardio-Renal Covariates

To evaluate metabolic and organ-specific correlates of inflammation, the following covariates were included:

- Age (years).
- Sex.
- Body mass index (BMI, kg/m^2^).
- Glycated hemoglobin (HbA1c, %).
- Triglyceride-to-HDL cholesterol ratio (TRIG/HDL).
- Estimated glomerular filtration rate (eGFR; mL/min/1.73 m^2^).
- Functional CKD (eGFR < 60 mL/min/1.73 m^2^).
- Composite ASCVD endpoint including documented diagnoses of ischemic heart disease, myocardial infarction, stroke, or peripheral arterial disease recorded in the electronic medical records.

The ASCVD composite variable was constructed from documented cardiovascular diagnoses recorded in the electronic medical records.

### 2.4. Statistical Analysis

Continuous variables were expressed as mean ± standard deviation (SD) or median with interquartile range (IQR), depending on distribution. Categorical variables were reported as counts and percentages.

Differences in CRP and ESR across diabetes duration strata were evaluated using the Kruskal–Wallis test due to non-normal distribution.

To investigate nonlinear associations between diabetes duration and CRP, quadratic regression models were constructed, including both duration and duration^2^ terms. Log-transformed CRP [log(CRP + 1)] was used as the dependent variable. Adjusted models included age, sex, BMI, HbA1c, and functional CKD as covariates.

Spearman correlation analyses were performed to assess associations between inflammatory markers and metabolic (BMI, TRIG/HDL, HbA1c) and renal parameters (eGFR, CKD status). Additional correlations between inflammatory markers and ASCVD were evaluated to explore cardio–renal–inflammatory interactions. Because renal dysfunction is strongly associated with both diabetes duration and systemic inflammation, functional CKD was included as an adjustment variable. However, CKD may also represent an intermediate pathway linking prolonged diabetes exposure with increased inflammatory burden; therefore, adjustment for CKD may partially attenuate the observed association.

All statistical tests were two-tailed, and statistical significance was defined as *p* < 0.05. Analyses were performed using IBM SPSS Statistics, Version 30 (IBM Corp., Armonk, NY, USA).

### 2.5. Sample Size Considerations

The study included 250 participants with complete inflammatory and clinical data. All eligible consecutive patients during the study period were included to maximize statistical power.

The number of participants per duration stratum (*n* = 52, 54, 96, and 48, respectively) provided adequate power to detect moderate effect sizes in non-parametric comparisons and regression models. Multivariable regression models satisfied conventional stability criteria, with sufficient observations relative to the number of predictors included.

### 2.6. Ethical Considerations

This study was observational and involved analysis of routinely collected clinical data. No experimental interventions were performed. All data were anonymized prior to statistical analysis to ensure confidentiality.

## 3. Results

### 3.1. Distribution of Inflammatory Markers Across Different Diabetes Duration Categories

A total of 250 adults with type 2 diabetes were stratified according to diabetes duration into four predefined categories: 0–4 years (*n* = 52), 5–9 years (*n* = 54), 10–14 years (*n* = 96), and ≥15 years (*n* = 48).

Given the non-normal distribution of inflammatory markers, C-reactive protein (CRP) and erythrocyte sedimentation rate (ESR) were analyzed using non-parametric statistics and reported as medians with interquartile ranges (IQR).

Median CRP values demonstrated a non-linear pattern across duration strata. CRP levels were higher in patients with 0–4 years of diabetes (median 0.62 mg/L; IQR 0.22–2.04) and in those with ≥15 years (median 0.77 mg/L; IQR 0.29–2.11), while intermediate groups showed lower median values (0.51 mg/L for 5–9 years and 0.43 mg/L for 10–14 years). Although this pattern suggested a U-shaped distribution, the overall difference across groups did not reach statistical significance (Kruskal–Wallis *p* = 0.063). Baseline characteristics of the overall cohort are presented in Table 1. Differences in baseline characteristics across diabetes-duration strata were explored descriptively but were not the primary focus of the present analysis.

In contrast, ESR demonstrated a statistically significant difference across duration strata (Kruskal–Wallis *p* = 0.002). Median ESR values were 15.0 mm/h (IQR 8.0–24.5) in the 0–4-year group, decreased to 10.5 mm/h (IQR 5.3–17.0) in the 5–9-year group, increased to 14.5 mm/h (IQR 9.0–22.3) in the 10–14-year group, and reached the highest levels in patients with ≥15 years of diabetes (median 20.0 mm/h; IQR 11.0–30.0).

**Figure 1:**
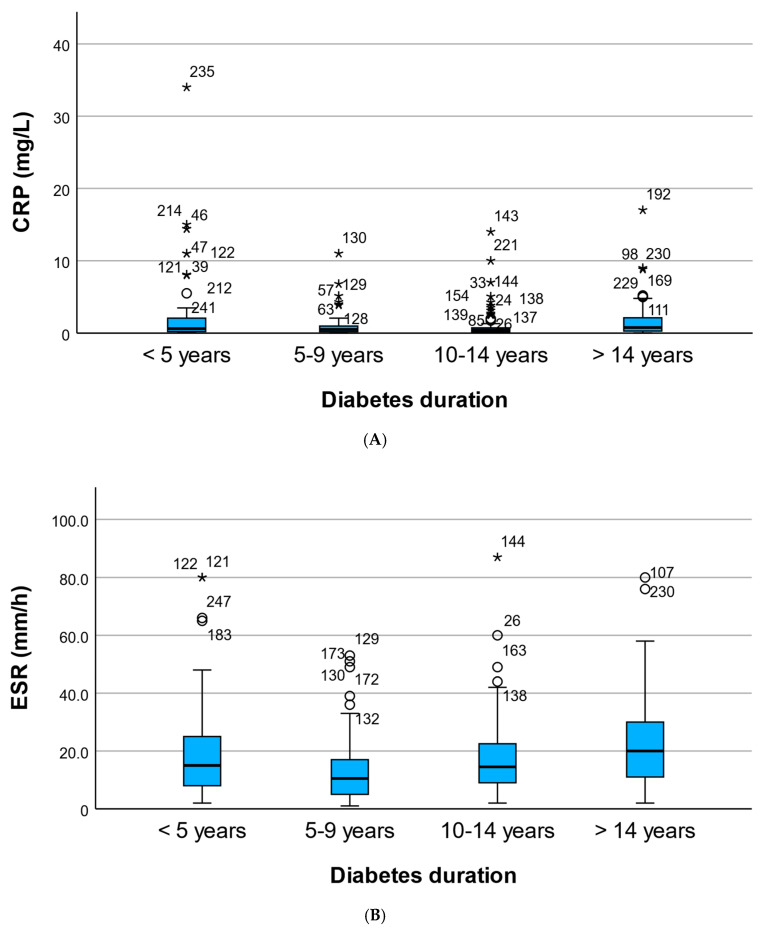
CRP and ESR across diabetes duration strata. (**A**) Boxplot illustrating the distribution of C-reactive protein (CRP, mg/L) across diabetes duration categories (0–4, 5–9, 10–14, and ≥15 years). A non-linear pattern is observed, with higher CRP levels in early-duration diabetes and in long-standing diabetes compared with intermediate-duration groups. (**B**) Boxplot illustrating the distribution of erythrocyte sedimentation rate (ESR, mm/h) across diabetes duration categories (0–4, 5–9, 10–14, and ≥15 years). ESR differs significantly across strata and shows higher values in long-standing diabetes, consistent with an increased inflammatory burden. Diabetes duration strata: 0–4, 5–9, 10–14, ≥15 years. * Asterisk (*) indicates extreme outliers, defined as values located more than 3 box-lengths (interquartile ranges) from the upper or lower edge of the box. ○ Hollow circles represent mild outliers, defined as values located between 1.5 and 3 box-lengths (interquartile ranges) from the box. Numbers displayed next to symbols denote the individual participant (case) identification numbers corresponding to those outlier values.

These findings suggest a possible U-shaped cross-sectional pattern characterized by relatively higher inflammatory levels in early and long-standing diabetes. However, this observation should be interpreted cautiously, as the group-wise comparison across duration strata did not reach conventional statistical significance (Figure 1).

### 3.2. Nonlinear Association Between Diabetes Duration and CRP

Given the non-normal distribution of CRP values, log-transformed CRP [log(CRP + 1)] was modeled as the dependent variable. To test for a nonlinear relationship between diabetes duration and inflammatory burden, both duration and its quadratic term (duration^2^) were included in regression models (Table 2).

In unadjusted analysis, both duration (β = −0.091, *p* < 0.001) and duration^2^ (β = 0.0039, *p* < 0.001) were statistically significant, confirming a nonlinear association between diabetes duration and CRP levels (R^2^ = 0.076).

A multivariable model adjusted for age, sex, body mass index (BMI), HbA1c, and functional CKD (eGFR < 60 mL/min/1.73 m^2^) demonstrated persistence of the nonlinear pattern. Duration remained negatively associated with log(CRP) (adjusted β = −0.079, *p* < 0.001), while the quadratic term remained independently significant (adjusted β = 0.0027, *p* < 0.001). The adjusted model explained 32.6% of the variance in log-transformed CRP (R^2^ = 0.326).

The negative linear term combined with the positive quadratic term indicates a U-shaped relationship, characterized by higher inflammatory burden at early stages of diabetes, lower levels during intermediate duration, and a subsequent increase in long-standing disease (Table 3).

**Figure 2:**
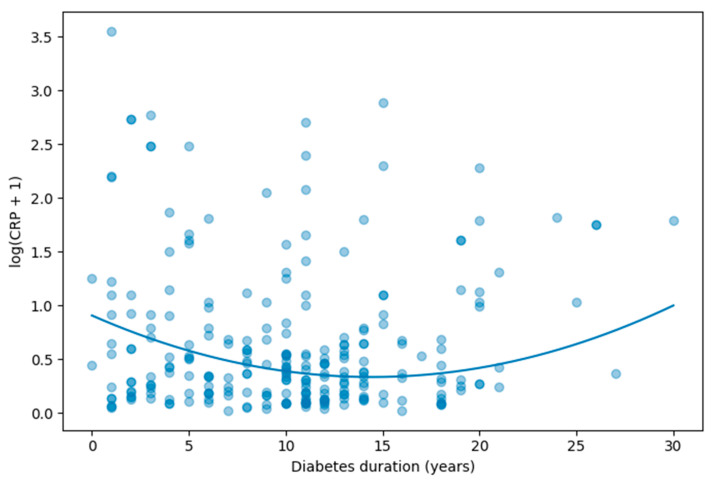
Nonlinear association between diabetes duration and CRP. Scatter plot illustrating log-transformed CRP values across diabetes duration, with superimposed quadratic regression curve adjusted for age, sex, BMI, HbA1c, and CKD status. The model demonstrates a U-shaped association, characterized by higher inflammatory burden in early and long-standing diabetes. Different shades of blue are used to improve visualization of overlapping observations; darker shades indicate areas with a higher concentration of data points, whereas lighter shades correspond to more isolated observations. The solid blue curve represents a locally weighted smoothing (LOESS) regression line, illustrating the overall trend in inflammatory status across increasing diabetes duration.

To visually illustrate the nonlinear relationship between diabetes duration and inflammatory burden, a scatter plot with superimposed quadratic regression curve was generated (Figure 2). As shown, log-transformed CRP values display a nonlinear cross-sectional pattern across increasing diabetes duration. Higher inflammatory levels are observed in early-stage diabetes, followed by a relative decline during intermediate duration, and a subsequent increase in long-standing disease. This graphical representation supports the statistical findings from the quadratic regression model and highlights the U-shaped cross-sectional pattern of inflammatory activity across the disease course.

### 3.3. Correlations Between CRP and Metabolic and Renal Parameters

To further explore the determinants of inflammatory burden, Spearman correlation analyses were performed between CRP and selected metabolic and renal variables.

CRP demonstrated a weak but statistically significant positive correlation with body mass index (BMI) (ρ = 0.151; *p* = 0.017), suggesting an association between adiposity and systemic inflammation.

A stronger positive correlation was observed between CRP and the triglyceride-to-HDL cholesterol ratio (TRIG/HDL) (ρ = 0.215; *p* < 0.001), supporting the link between atherogenic dyslipidemia and inflammatory activation.

No significant correlation was observed between CRP and HbA1c (ρ = −0.079; *p* = 0.211), indicating that inflammatory burden in this cohort was not directly related to short-term glycemic control.

Importantly, CRP showed a moderate inverse correlation with estimated glomerular filtration rate (eGFR) (ρ = −0.333; *p* < 0.001), indicating higher inflammatory levels in patients with reduced kidney function.

These findings suggest that systemic inflammation in type 2 diabetes is more strongly associated with adiposity, dyslipidemia, and renal impairment than with current glycemic control (Table 4).

### 3.4. Cardio–Renal–Inflammatory Interactions

To explore the interplay between cardiovascular burden, renal dysfunction, and systemic inflammation, Spearman correlation analyses were performed between inflammatory markers (CRP and ESR) and both ASCVD status and functional CKD (eGFR < 60 mL/min/1.73 m^2^).

CRP demonstrated a weak, borderline association with ASCVD (ρ = 0.120; *p* = 0.058), suggesting a modest relationship between systemic inflammation and established cardiovascular disease.

In contrast, ESR showed a statistically significant positive correlation with ASCVD (ρ = 0.189; *p* = 0.003), indicating higher inflammatory levels in patients with manifest atherosclerotic disease.

More strikingly, both inflammatory markers demonstrated strong associations with renal impairment. CRP correlated positively with functional CKD (ρ = 0.350; *p* < 0.001), while ESR showed an even stronger association (ρ = 0.429; *p* < 0.001).

These findings suggest that systemic inflammation in this cohort is more strongly linked to renal dysfunction than to overt cardiovascular disease (Table 5).

### 3.5. Adiposity–Dyslipidemia Axis as Determinants of Inflammatory Burden

To further investigate whether inflammatory burden is primarily driven by metabolic phenotype rather than glycemic exposure, a multivariable linear regression model was constructed using log-transformed CRP [log(CRP + 1)] as the dependent variable.

The model included body mass index (BMI), triglyceride-to-HDL cholesterol ratio (TRIG/HDL), HbA1c, diabetes duration, age, sex, and functional CKD (eGFR < 60 mL/min/1.73 m^2^).

In the fully adjusted model (R^2^ = 0.292), BMI emerged as an independent predictor of inflammatory burden (adjusted β = 0.018; 95% CI 0.006 to 0.030; *p* = 0.003). Each 1 kg/m^2^ increase in BMI was associated with a significant increase in log-transformed CRP.

Functional CKD demonstrated the strongest association with inflammation (adjusted β = 0.748; 95% CI 0.569 to 0.926; *p* < 0.001), confirming the central role of renal impairment in amplifying systemic inflammatory activity.

Diabetes duration remained independently associated with CRP (adjusted β = −0.018; 95% CI −0.032 to −0.005; *p* = 0.008), supporting the previously identified nonlinear cross-sectional pattern.

In contrast, TRIG/HDL ratio (β = 0.0013; *p* = 0.635) and HbA1c (β = −0.028; *p* = 0.226) were not independently associated with CRP after adjustment. Age and sex were also not significant predictors.

These findings indicate that adiposity and renal dysfunction exert stronger independent effects on inflammatory burden than current glycemic control or atherogenic dyslipidemia when considered simultaneously (Table 6).

## 4. Discussion

In this real-world cohort of adults with type 2 diabetes, inflammatory burden demonstrated a nonlinear association with diabetes duration, characterized by higher levels in early-stage disease and again in long-standing diabetes. This nonlinear cross-sectional pattern was particularly evident for CRP in quadratic regression modeling and for ESR in both descriptive and inferential analyses.

### 4.1. A Biphasic Inflammatory Pattern Across Diabetes Duration

Our findings suggest that systemic inflammation in this cohort of patients with type 2 diabetes does not appear to follow a simple monotonic cross-sectional pattern across diabetes duration strata. CRP levels were elevated in patients with short disease duration (0–4 years), decreased during intermediate stages (5–14 years), and increased again in long-standing diabetes (≥15 years). The quadratic regression model confirmed this U-shaped association even after adjustment for age, BMI, HbA1c, and CKD status.

This pattern may reflect different clinical and pathophysiological profiles across diabetes duration strata. Early-stage diabetes is often characterized by active insulin resistance, adipose tissue dysfunction, and metabolic inflammation driven by cytokines such as IL-6 and TNF-α. During intermediate stages, improved metabolic control or treatment intensification may contribute to lower inflammatory levels in intermediate-duration groups, although medication exposure was not formally evaluated in the present analysis [15,16,17].

In contrast, long-standing diabetes is frequently accompanied by microvascular and macrovascular complications, renal dysfunction, sarcopenia, and frailty, all of which may contribute to renewed systemic inflammatory activation [15,18,19,20].

### 4.2. Inflammation Is More Closely Linked to Renal Dysfunction than Cardiovascular Disease

One of the most important findings of this study is that inflammatory markers were more strongly associated with renal impairment than with established cardiovascular disease.

CRP and ESR demonstrated moderate positive correlations with functional CKD (ρ = 0.350 and ρ = 0.429, respectively; *p* < 0.001), whereas their association with ASCVD was weaker and, in the case of CRP, borderline significant. Because renal dysfunction may represent both a confounding factor and an intermediate pathway linking prolonged diabetes exposure with systemic inflammation, interpretation of adjusted analyses should consider the potential mediating role of CKD.

This suggests that renal dysfunction may represent a key amplifier of systemic inflammation in long-standing diabetes. Reduced glomerular filtration leads to impaired clearance of pro-inflammatory mediators, accumulation of uremic toxins, and persistent immune activation. The stronger association with CKD compared to ASCVD supports the concept of a cardio–renal–inflammatory axis in which renal decline acts as a central driver of inflammatory burden [21,22,23,24].

### 4.3. Inflammation and Metabolic Correlates

Inflammatory burden correlated significantly with BMI and the triglyceride-to-HDL cholesterol ratio, but not with HbA1c. These findings indicate that inflammation in this cohort is more closely linked to adiposity and dyslipidemia than to short-term glycemic control [25,26].

The lack of significant association between CRP and HbA1c suggests that systemic inflammation may be influenced more by chronic metabolic milieu and organ damage than by current glycemic status. This reinforces the concept that inflammation represents a broader cardiometabolic phenotype rather than a direct marker of glucose levels [27,28].

### 4.4. Integration with the Cardiovascular and Renal Analyses

When integrated with the companion analyses of cardiovascular and renal risk across diabetes duration, a coherent pattern emerges:

- Cardiovascular burden appears largely influenced by chronological aging.
- Renal dysfunction shows a strong and independent association with cumulative diabetes exposure.
- Inflammatory burden demonstrates a nonlinear pattern and is more strongly linked to renal impairment than to ASCVD.

Together, these findings suggest that prolonged diabetes duration contributes to renal decline, which in turn may amplify systemic inflammation. This pathway may partially explain the increased vulnerability observed in long-standing diabetes [29,30,31].

### 4.5. Clinical Implications

The observation of a possible nonlinear inflammatory pattern may have potential clinical implications. Elevated inflammation at diagnosis may reflect an aggressive metabolic phenotype that warrants early intensive lifestyle and pharmacologic intervention. In long-standing diabetes, increased inflammatory burden may signal advanced organ damage, particularly renal dysfunction, and may identify patients at higher risk of adverse outcomes.

Monitoring inflammatory markers such as CRP and ESR may provide additional insight into disease stage and organ involvement beyond traditional glycemic metrics [32,33].

Given the cross-sectional design, the observed pattern should not be interpreted as a within-person trajectory of inflammatory burden over time. Instead, the findings represent differences across diabetes duration strata within this cohort. Longitudinal studies are required to determine whether similar nonlinear patterns occur within individuals as diabetes progresses.

### 4.6. Limitations

This study is cross-sectional and cannot establish causal relationships. Inflammatory markers were limited to CRP and ESR, and more specific cytokine profiling was not available. Residual confounding cannot be excluded despite multivariable adjustment. In particular, medication exposure—including antidiabetic therapies, lipid-lowering agents such as statins, and other treatments with potential anti-inflammatory effects—may influence CRP, ESR, and metabolic parameters but was not systematically incorporated into the statistical models. Additionally, the absence of longitudinal follow-up precludes evaluation of inflammatory trajectories over time.

An important limitation relates to the cross-sectional design and potential survivor or selection bias. Patients with longer diabetes duration represent a selective population who have survived earlier disease stages and may differ systematically in treatment intensity, clinical monitoring, and comorbidity burden. Such survivor effects can generate non-linear cross-sectional patterns that do not necessarily reflect within-person trajectories over time. Therefore, the observed U-shaped association across duration strata should be interpreted cautiously and confirmed in longitudinal cohorts.

In addition, chronic inflammatory conditions, smoking status, liver disease, malignancy, and other comorbidities that may influence inflammatory markers were not systematically recorded in the database and therefore could not be fully controlled for in the analysis. The unequal distribution of participants across diabetes-duration strata may have influenced statistical power and the stability of subgroup comparisons.

ASCVD was analyzed as a composite endpoint including ischemic heart disease, myocardial infarction, stroke, and peripheral arterial disease. Because individual cardiovascular components were not analyzed separately, potential differences between specific ASCVD manifestations could not be evaluated.

## 5. Conclusions

In this real-world cohort of adults with type 2 diabetes, inflammatory burden demonstrated a nonlinear association with diabetes duration, characterized by elevated levels in early-stage disease and again in long-standing diabetes. Quadratic regression modeling confirmed a U-shaped relationship between diabetes duration and CRP, independent of age, adiposity, glycemic control, and renal function.

Importantly, inflammatory markers were more strongly associated with renal impairment than with established cardiovascular disease. Both CRP and ESR showed robust correlations with functional CKD, suggesting that renal dysfunction may represent a key driver of systemic inflammation in prolonged diabetes exposure.

These findings indicate that inflammation in type 2 diabetes reflects distinct pathophysiological duration strata: an early metabolically active inflammatory state and a later stage linked to cumulative organ damage, particularly renal decline. Monitoring inflammatory markers may provide additional insight into inflammatory burden in patients with type 2 diabetes, although these findings should be considered exploratory and require confirmation in longitudinal studies.

Future longitudinal studies are needed to clarify whether modulation of inflammatory burden—particularly in patients with long-standing diabetes and declining kidney function—can modify clinical outcomes.

## Figures and Tables

**Table 1:** Baseline characteristics of the study population (*n* = 250).

Variable	Value
Age (years)	61.8 ± 11.3
Male sex, *n* (%)	128 (51.2%)
BMI (kg/m^2^)	32.9 ± 6.0
HbA1c (%)	7.86 ± 1.67
Chronic kidney disease (eGFR < 60 mL/min/1.73 m^2^), *n* (%)	60 (24.0%)
ASCVD (composite endpoint), *n* (%)	213 (85.2%)
Antidiabetic therapy	
Oral antidiabetic drugs, *n* (%)	147 (58.8%)
Metformin, *n* (%)	147 (58.8%)
Sulfonylurea, *n* (%)	64 (25.6%)
DPP-4 inhibitors, *n* (%)	15 (6.0%)
GLP-1 receptor agonists, *n* (%)	72 (28.8%)
SGLT2 inhibitors, *n* (%)	69 (27.6%)

Continuous variables are presented as mean ± standard deviation, while categorical variables are expressed as number (percentage). ASCVD composite endpoint included ischemic heart disease, myocardial infarction, stroke, or peripheral arterial disease.

**Table 2:** Inflammatory markers across diabetes duration strata.

Duration Group	*N*	CRP Median (IQR), mg/L	ESR Median (IQR), mm/h
0–4 years	52	0.62 (0.22–2.04)	15.0 (8.0–24.5)
5–9 years	54	0.51 (0.20–0.98)	10.5 (5.3–17.0)
10–14 years	96	0.43 (0.17–0.73)	14.5 (9.0–22.3)
≥15 years	48	0.77 (0.29–2.11)	20.0 (11.0–30.0)

CRP, C-reactive protein; ESR, erythrocyte sedimentation rate; IQR, interquartile range; *N*, number of participants. Data are presented as median (interquartile range). Differences across duration strata were assessed using the Kruskal–Wallis test (CRP *p* = 0.063, ESR *p* = 0.002).

**Table 3:** Adjusted quadratic regression model for log-transformed CRP.

Term	Adjusted β	95% CI	*p*-Value
Duration (years)	−0.079	−0.116 to −0.043	<0.001
Duration^2^	0.0027	0.0012 to 0.0043	<0.001

CRP, C-reactive protein; CI, confidence interval. Log-transformed CRP [log(CRP + 1)] was used as the dependent variable. The model was adjusted for age, sex, body mass index, HbA1c, and functional chronic kidney disease (eGFR < 60 mL/min/1.73 m^2^). Model R^2^ = 0.326. The quadratic term represents the squared diabetes duration variable included to assess nonlinear association.

**Table 4:** Spearman correlations between CRP and selected clinical parameters.

Variable	Spearman ρ	*p*-Value
BMI	0.151	0.017
HbA1c	−0.079	0.211
TRIG/HDL ratio	0.215	<0.001
eGFR	−0.333	<0.001

CRP, C-reactive protein; BMI, body mass index; HbA1c, glycated hemoglobin; TRIG/HDL, triglyceride-to-high-density lipoprotein cholesterol ratio; eGFR, estimated glomerular filtration rate. Spearman’s rank correlation coefficients (ρ) are presented.

**Table 5:** Spearman correlations between inflammatory markers and cardio-renal outcomes.

Outcome	CRP (ρ)	*p*-Value	ESR (ρ)	*p*-Value
ASCVD	0.120	0.058	0.189	0.003
CKD (eGFR < 60)	0.350	<0.001	0.429	<0.001

CRP, C-reactive protein; ESR, erythrocyte sedimentation rate; ASCVD, atherosclerotic cardiovascular disease; CKD, chronic kidney disease; eGFR, estimated glomerular filtration rate. Spearman’s rank correlation coefficients (ρ) are presented. CKD was defined as eGFR < 60 mL/min/1.73 m^2^.

**Table 6:** Multivariable regression model for log-transformed CRP.

Variable	Adjusted β	95% CI	*p*-Value
BMI (kg/m^2^)	0.018	0.006 to 0.030	0.003
TRIG/HDL ratio	0.0013	−0.004 to 0.007	0.635
HbA1c (%)	−0.028	−0.072 to 0.017	0.226
Diabetes duration (years)	−0.018	−0.032 to −0.005	0.008
Age (years)	0.006	−0.001 to 0.013	0.079
Male sex	−0.006	−0.149 to 0.137	0.931
CKD (eGFR < 60)	0.748	0.569 to 0.926	<0.001

CRP, C-reactive protein; BMI, body mass index; TRIG/HDL, triglyceride-to-high-density lipoprotein cholesterol ratio; HbA1c, glycated hemoglobin; CKD, chronic kidney disease; eGFR, estimated glomerular filtration rate; CI, confidence interval. Log-transformed CRP [log(CRP + 1)] was used as the dependent variable. CKD was defined as eGFR < 60 mL/min/1.73 m^2^. Model R^2^ = 0.292.

## Data Availability

The original contributions presented in this study are included in this article. Further inquiries can be directed to the first authors.

## Abbreviations

The following abbreviations are used in this manuscript:

ASCVD	Atherosclerotic cardiovascular disease
BMI	Body mass index
CI	Confidence interval
CKD	Chronic kidney disease
CRP	C-reactive protein
eGFR	Estimated glomerular filtration rate
ESR	Erythrocyte sedimentation rate
HbA1c	Glycated hemoglobin
IQR	Interquartile range
TRIG/HDL	Triglyceride-to-high-density lipoprotein cholesterol ratio
T2DM	Type 2 diabetes mellitus
